# An observational study of pain severity, cannabis use, and benefit expenditures in work disability

**DOI:** 10.17269/s41997-023-00821-1

**Published:** 2023-10-16

**Authors:** Cameron A. Mustard, Christa Orchard, Kathleen G. Dobson, Nancy Carnide, Peter M. Smith

**Affiliations:** 1 Institute for Work & Health, Toronto, ON Canada; 2 Dalla Lana School of Public Health, University of Toronto, Toronto, ON Canada

**Keywords:** Cannabis, Pain severity, Occupational health, Disability insurance, Public health, Cannabis, gravité de la douleur, santé au travail, assurance invalidité, santé publique

## Abstract

**Objective:**

This study pools two cohorts of workers in Ontario interviewed 18 months following a disabling work-related injury to estimate the association between pain severity, cannabis use, and disability benefit expenditures.

**Methods:**

Among 1650 workers, disability benefit expenditures obtained from administrative records were combined with self-reported measures of pain symptoms and cannabis use. Disability benefit expenditures comprised wage replacement benefits and expenditures on healthcare services.

**Results:**

Past-year cannabis use was reported by 31% of participants, with approximately one third of cannabis use attributed to the treatment of conditions arising from the work-related injury. Condition-related cannabis use was elevated among the 34% of participants reporting severe pain symptoms. In regression models adjusted for age, sex, nature of injury, opioid prescription, and pre-injury chronic conditions, participants reporting condition-related cannabis use had equivalent wage replacement benefit expenditures (*β* = 0.254, ns) and higher healthcare benefit expenditures (*β* = 0.433, *p* = 0.012) compared to participants who did not use cannabis. Participants reporting cannabis use unrelated to conditions arising from their work-related injury had lower wage replacement benefit expenditures (*β* =  − 0.309,* p* = 0.002) and equivalent healthcare benefit expenditures (*β* =  − 0.251, ns) compared to participants not using cannabis.

**Conclusion:**

This novel study of workers’ compensation claimants interviewed at 18 months post-injury did not observe a substantial relationship between cannabis use and disability benefit expenditures, suggesting that neither harm nor significant benefit is associated with cannabis use. These findings contribute to understanding the potential benefits and risks associated with cannabis use in settings that have legalized cannabis use.

## Résumé

**Objectif:**

Cette étude regroupe deux cohortes de travailleurs et travailleuses de l’Ontario interviewés 18 mois après un accident de travail invalidant; elle vise à estimer l’association entre la gravité de la douleur, la consommation de cannabis et les dépenses en prestations d’invalidité.

**Méthode:**

Les dépenses en prestations d’invalidité de 1 650 travailleurs et travailleuses, obtenues en consultant les dossiers administratifs, ont été combinées aux indicateurs autodéclarés de symptômes de douleur et de consommation de cannabis. Les dépenses en prestations d’invalidité englobaient les prestations de remplacement du salaire et les dépenses en services de soins de santé.

**Résultats:**

Une consommation de cannabis au cours de la dernière année a été déclarée par 31 % des participants; environ le tiers de cette consommation de cannabis était imputée au traitement d’affections causées par l’accident de travail. La consommation de cannabis liée à une affection était élevée chez les 34 % de participants ayant déclaré de graves symptômes de douleur. Selon nos modèles de régression ajustés selon l’âge, le sexe, la nature de la blessure, la prescription d’opioïdes et l’existence d’états chroniques avant l’accident, pour les participants ayant déclaré une consommation de cannabis liée à une affection, les dépenses en prestations de remplacement du salaire étaient équivalentes (β = 0,254, ns) et les dépenses en prestations de soins de santé étaient supérieures (β = 0,433, *p* = 0,012) à celles des participants n’ayant pas consommé de cannabis. Pour les participants ayant déclaré une consommation de cannabis sans rapport avec des affections causées par leur accident de travail, les dépenses en prestations de remplacement du salaire étaient inférieures (β = -0,309, *p* = 0,002) et les dépenses en prestations de soins de santé étaient équivalentes (β = -0,251, ns) à celles des participants n’ayant pas consommé de cannabis.

**Conclusion:**

Cette étude novatrice menée auprès de demandeurs d’indemnités interviewés 18 mois après leur accident n’a pas observé de relation importante entre la consommation de cannabis et les dépenses en prestations d’invalidité, ce qui semble indiquer que ni des préjudices, ni des avantages significatifs ne sont associés à la consommation de cannabis. Ces constats contribuent à la compréhension des avantages et des risques qui pourraient être associés à la consommation de cannabis dans les milieux où cette consommation est légale.

## Introduction

Among working-age adults in North America, one of every six injuries requiring medical attention arises from exposures at work (Chambers et al., 2015). Injuries and illnesses attributed to work exposures have substantial societal economic impact, with the approximately 35% of work-related injuries and illnesses that result in periods of disability and work absence responsible for the majority of this economic burden (Leigh, 2011). Work-related injuries requiring work absence can also result in consequences to workers’ long-term health, including deficits in physical health (Baragaba et al., 2016), mental health (O’Hagan et al., 2012), and the incidence of chronic conditions (Dobson et al., 2023).

Among the consequences of traumatic injury is a substantial incidence of persistent or chronic pain (Alkassabi et al., 2022; Pierik et al., 2016; Rivara et al., 2008; Rosenbloom et al., 2016). Recognition of the etiologic role of traumatic injury in the population burden of chronic pain has led to proposals to revise the classification of chronic pain in ICD-11 to include ‘chronic post-surgical and posttraumatic pain’ as one of seven etiologic categories (Treede et al., 2019). In a recent analysis of the cohort of workers described in this current study, 24.9% reported severe pain intensity with substantial functional impairment 18 months after a disabling work-related injury (Dobson et al., 2022). The prevalence of severe pain symptoms in this cohort is approximately six times higher than in North American adult populations (Pitcher et al., 2019).

Population monitoring of cannabis use in North America has consistently found that 25–35% of adults using cannabis are doing so for therapeutic purposes (Carnide et al., 2021; Rotermann and Pagé, 2018; Leung et al., 2022; Schauer et al., 2022). In both population-based studies and studies of adults with chronic pain, the use of cannabis for the management of pain symptoms has been commonly reported (Godbout-Parent et al., 2022; Leung et al., 2022). As is typical in these observational studies of therapeutic cannabis use, underlying medical conditions, durations of the conditions, and the age profile of cohort members are heterogeneous.

The efficacy of cannabis for the treatment of chronic noncancer pain has been evaluated in a substantial number of randomized controlled trials and observational studies. A recent systematic review and meta-analysis of more than 100 controlled and observational studies, while noting some evidence for therapeutic efficacy in neuropathic pain, concluded that ‘it seems unlikely that cannabinoids are highly effective medicines for chronic noncancer pain’, also noting limited evidence for improvement in emotional and physical functioning (Stockings et al., 2018). Consistent with the conclusions of this review, a 4-year prospective cohort study of 1500 chronic pain patients with opioid prescriptions observed that among the 24% of participants who used cannabis, there was no evidence of a benefit of cannabis use on pain severity, pain interference, or opioid discontinuation (Campbell et al., 2018). In contrast, a meta-analysis of six observational studies of a minimum of 26 weeks duration did report a non-significant mean pain reduction of 1.75 on a 0–10 scale with 20% of patients reporting pain relief of 50% or greater (Bialas, 2022).

In addition to the uncertain evidence for therapeutic benefit of cannabis use in the management of chronic pain symptoms, the relationship between therapeutic cannabis use and health care utilization is not well described. A large prospective observational study of more than 9000 adults with medical authorization for cannabis use matched on age, sex, and prevalent chronic conditions to persons without medical cannabis authorization did not detect important differences in physician visits, hospitalization, or emergency department visits over a 12-month period following medical authorization (Eurich et al., 2020). While additional replication studies are indicated, this study suggests that therapeutic cannabis use does not modify the overall utilization of health care services.

This study pools two longitudinal cohorts of disabled workers to estimate the association between the severity of pain symptoms 18 months following a disabling work-related injury, cannabis use, and disability benefit expenditures. Among disabled workers in these cohorts, cannabis use and disability benefit expenditures are elevated among participants with severe pain symptoms (Dobson et al., 2022). As described below, the cohort design oversampled disability benefit recipients with longer duration disability episodes and incorporated measures obtained from administrative records of the workers’ compensation authority with measures obtained from interviews with study subjects 18 months following the incidence of disabling injury or illness. The study did not have specific a priori hypotheses concerning the magnitude or direction of potential associations between cannabis use and disability benefit expenditures.

## Methods

### Setting

Of the seven million labour force participants in Ontario, Canada, approximately 70% are employed by organizations that have a mandatory obligation to obtain work disability insurance coverage from the publicly administered, single-payer workers’ compensation insurance authority, the Workplace Safety & Insurance Board (WSIB). The WSIB administers benefits to workers eligible for coverage, reimbursing costs of medical care services and providing wage replacement benefits for workers whose recovery from a work-related injury or illness requires absence from work. In 2018, the WSIB administered benefits for 200,000 compensation claims, of which 67,000 were claims resulting in lost-time from work.

### Study design

Prospective observational cohort.

### Sample

This study pools information from two cohorts of workers in Ontario, Canada, participating in the Ontario Life After Work Injury Study (OLAWIS1, *N* = 1132; OLAWIS2, *n* = 700) who were disabled by a physical work injury or illness and received wage replacement benefits for an absence from work (Mustard et al., 2021). The sample frame for participant recruitment was based on the universe of accepted compensation claims for wage replacement benefits administered by the WSIB. Representatives of the WSIB prepared stratified samples of the sample frame following specifications provided by the project study team. To obtain sufficient representation of more serious and complex disability episodes, participants with longer wage replacement durations of 3 months or greater were oversampled, representing 58.9% of the cohort. The short-duration sample (wage replacement benefits of 1 day to 3 months) represents 85% of all wage replacement claimants in this setting and the long-duration sample (more than 3 months) represents 15% of all wage replacement claimants. Both cohorts comprised Ontario workers > 18 years old, who were employed by an insured employer, able to conduct an interview in English or French, and had experienced a work-related injury or occupational disease that resulted in a WSIB accepted compensation claim for wage replacement benefits.

Participant recruitment for OLAWIS1 occurred between June 2019 and March 2020, among workers disabled by a work-related injury in the period January to October 2018. From randomly sampled monthly quotas of lost-time claimants meeting eligibility criteria, 2816 randomly sampled claimants were contacted, of whom 1674 (59.4%) agreed to share their contact information with the research team. Subsequently, a survey services contractor contacted consenting workers and conducted an interviewer-administered telephone interview, completing interviews with 1132 claimants (40.2% of eligible claimants and 87.8% of eligible claimants successfully contacted). Among participants, 358 (31.6%) were in the short-duration claim sample, and 774 (68.3%) were in the long-duration claim sample. More details on the OLAWIS1 study cohort may be found elsewhere (Mustard et al., 2021).

Participant recruitment for OLAWIS2 occurred over the period September to November 2021. From a census of all claimants disabled by a work-related injury or illness in January or February 2020, 2309 randomly sampled claimants were contacted, among whom a survey services contractor completed interviews with 700 (30.3% of eligible claimants). Among OLAWIS2 participants, 395 (56.4%) were in the short-duration claim sample, and 305 (43.6%) were in the long-duration claim sample.

The present study included 1650 cohort members who provided information on pain symptoms and cannabis use at 18 months and who consented to link their survey responses to an administrative record of their WSIB compensation claim (OLAWIS1, *n* = 1062 (94%); OLAWIS2, *n* = 588 (84%)). Ethics review was conducted by the Health Sciences Research Ethics Board at the University of Toronto (Protocol 37525 and 41560).

### Data sources

The primary outcome measures, wage replacement benefit expenditures and healthcare benefit expenditures, were obtained from WSIB administrative records. These administrative records were also the source of information on the nature of injury and injury event, the workers’ occupation and geographic location, and the employer’s size and economic sector. An interviewer-administered telephone questionnaire 18 months after the original injury obtained detailed information on additional demographic, health, and employment characteristics.

### Benefit expenditure measures

Two measures of benefit expenditure (Canadian dollars) were obtained from WSIB administrative records for each cohort member over the 18-month follow-up period: (1) cumulative dollars of benefits provided to workers for wage replacement benefits during their absence from work and (2) cumulative dollars of health care services reimbursed by the WSIB for treatment of the work-related condition.

### Cannabis use

Participants in the interviewer-administered survey were asked a series of questions about cannabis use including lifetime use and use in the past 12 months. Participants reporting use in the past 12 months were asked about the frequency of use, whether use was for therapeutic purposes, and if so, was the cannabis use medically authorized (Carnide et al., 2021). Participants reporting past-year cannabis use were also asked if they were using cannabis in part for the treatment of conditions arising from their work-related injury. Participants who replied in the affirmative to this statement were classified as ‘condition-related cannabis use’. All other participants reporting past-year cannabis use were classified as ‘non-condition-related cannabis use’.

### Severity of pain symptoms

Interview participants were asked two questions related to pain symptoms. A pain interference question asked: “During the past 4 weeks, how much did pain interfere with your normal work (including both work outside the home and housework)?”, in which participants could respond: not at all, a little bit, moderately, quite a bit, or extremely (Hays et al., 1993). Participants indicating at least a little pain interference were then asked, “On a scale from 0 to 10, how would you rate your pain due to your injury at the present time (that is right now), where 0 is no pain and 10 is pain as bad as it could be?” (Flaherty, 1996).

To align with the evolving standard for the classification of persistent pain (Dahlhamer et al., 2018), three pain severity groups were defined based on pain symptoms over the past 4 weeks: (1) no pain, if participants responded that pain did not interfere with their normal activities; (2) mild pain with an unlikely or low impact on functional impairment, if participants reported that their pain interference was “a little bit”, or “moderate”, and their pain intensity score was less than 6/10; and (3) severe pain in which functional impairment was likely, if respondents reported pain interference of “quite a bit”, “extremely”, or their pain interference was “moderate” but pain intensity score was greater or equal to 6/10 (Dobson et al., 2022).

### Demographic, work, health, and injury factors

Participants were asked about demographic, work, health-related, and injury-related factors when interviewed at 18 months post-injury. Demographic factors included participant age, sex, highest level of education, country of birth, household income, and industry at time of injury. Participants were asked about the prevalence of five physician-diagnosed chronic conditions prior to injury (back problems, arthritis, migraine disorder, mood disorder, high blood pressure). Additionally, participants were also asked if they were still receiving services from the WSIB, if they experienced financial difficulties during their work absence, and their current employment status. Respondents were asked about their healthcare experience including currently receiving healthcare for their injury and perceptions of difficulties in accessing or receiving healthcare services. Finally, participants were asked about their health status at 18 months post-injury, including measures of overall physical and mental health and prescription opioid use. Measures of self-rated health status and self-rated mental health status were administered along with the SF-12 scale (Ware et al., 1996) and the Kessler-6 screening tool for mental disorders (Kessler et al., 2010). Information on the nature of injury resulting in work disability was obtained from administrative records.

### Analytic methods

Descriptive statistics reporting the distribution of demographic, employment, and health status measures were tabulated, stratified by past-year cannabis use status. Descriptive analyses were not weighted to account for the oversampling of longer duration disability episodes.

The wage replacement expenditure and health care expenditure information used in this study are strongly right skewed and do not meet the assumptions of a normal distribution. To describe average benefit expenditures by cannabis use status, dollar values above the 95^th^ percentile were truncated. To evaluate the association between cannabis use and benefit expenditures, log-transformed, untruncated benefit expenditure data were analyzed in ordinary least square regression models. We conducted a complete-case analysis, excluding observations missing information on one or more explanatory variables (pain symptom information was missing for 7 observations, benefit expenditure information was missing for 28 observations, and information was missing for 65 observations incorporated in multivariable analyses). Regression models, run separately for wage replacement benefits and for health care expenditures, were conducted in three steps. The initial models estimated the simple bivariate association between cannabis use and expenditures and, separately, pain severity and expenditures. The second model estimated associations with both cannabis use and pain severity in the same regression. The final model included adjustments for characteristics measured at the time of cohort inception, specifically age, sex, nature of injury (grouped in six categories), pre-injury self-reported chronic conditions, and membership in the OLAWIS1 or OLAWIS2 cohorts. These models also included a measure of past-year prescription opioid use obtained from the interview conducted 18 months post-injury. To aid the reader in interpretation, the exponent of the regression beta coefficients represents the percent difference in benefit expenditure between the strata and the reference group. For example, for pain severity, the two beta coefficients represent the relative percent difference in benefit expenditure between participants with mild pain (functional impairment unlikely) vs. no pain, and severe pain (functional impairment likely) vs. no pain. To evaluate the influence of the sample design, which over-sampled participants with longer duration disability episodes, regression analyses were replicated applying sample weights. All analyses were completed in SAS version 9.4.

## Results

The demographic, occupational, and health characteristics of the two OLAWIS cohorts were broadly equivalent, in terms of attributes measured at both cohort inception and at the 18-month follow-up interview. A total of 68.6% of participants reported no cannabis use in the past 12 months, 19.9% reported use unrelated to conditions associated with the work-related injury, and 11.5% reported use to manage conditions associated with the work-related injury (36% of all participants reporting cannabis use in the past 12 months) (Table 1). Condition-related cannabis use was moderately higher in the OLAWIS1 cohort compared to the OLAWIS2 cohort (13.1% vs 8.5%). Among participants reporting past-year cannabis use, 21.4% reported medical authorization and 30.0% reported daily use.

**Table 1:** OLAWIS cohort demographic, work, and health characteristics at cohort inception, by past-year cannabis use

	All respondents	Past-year cannabis use			
		No use	Non-condition-related use	Condition-related use	*P* value
*N*	1650	1132	328	190	
Row percent, unweighted (weighted)	100.0	68.7 (68.2)	19.9 (21.7)	11.5 (10.0)	
Short duration sample, *n* (%)	672 (41.4%)	456 (40.1%)	163 (50.5%)	53 (29.1%)	< 0.001
Long duration sample, *n* (%)	950 (58.6%)	661 (59.2%)	160 (49.5%)	129 (70.8%)	
Age, mean (SD)	47.0 (13.0)	49.3 (12.3)	41.6 (13.6)	42.6 (12.5)	< 0.0001
Less than 30 years old	223 (13.5)	107 (9.5%)	79 (24.1%)	37 (19.5%)	< 0.0001
30–39 years old	292 (17.7)	156 (13.8%)	84 (25.6%)	52 (27.4%)	
40–49 years old	317 (19.2)	228 (20.1%)	59 (18.0%)	30 (15.8%)	
50–59 years old	524 (31.8)	406 (35.9%)	67 (20.4%)	51 (26.8%)	
Over 60 years old	294 (17.8)	235 (20.8%)	39 (11.9%)	20 (10.5%)	
Female sex, *n* (%)	766 (46.4)	560 (49.5%)	126 (38.4%)	80 (42.1%)	0.0027
Highest level of education,* n* (%)					
High school diploma or less	464 (28.2)	304 (27.0%)	95 (29.1%)	65 (34.2%)	0.0337
College degree or trade certification	799 (48.6)	542 (48.1%)	161 (49.4%)	96 (50.5%)	
Undergraduate or graduate degree	380 (23.1)	281 (24.9%)	70 (21.5%)	29 (15.3%)	
Country of birth, outside Canada, *n* (%)	373 (22.7)	317 (28.2%)	36 (11.0%)	20 (10.5%)	< 0.0001
Household income, *n* (%)^*^					
< $40 K	197 (13.5)	122 (12.2%)	41 (14.3%)	34 (19.7%)	0.0210
$40–69 K	368 (25.2)	264 (26.3%)	55 (19.2%)	49 (28.3%)	
$70–99 K	326 (22.3)	224 (22.3%)	68 (23.8%)	34 (19.7%)	
$100–129 K	266 (18.2)	174 (17.4)	62 (21.7%)	30 (17.3%)	
≥ $130 K	305 (20.9)	219 (21.8%)	60 (21.0%)	26 (15.0%)	
Industry at time of claim, *n* (%)					
Health care and social assistance	255 (15.5)	182 (16.1%)	42 (12.8%)	31 (16.3%)	0.0044
Construction, utilities, mining, agriculture, forestry	227 (13.8)	132 (11.7%)	55 (16.8%)	40 (21.1%)	
Transportation and warehousing	241 (14.6)	184 (16.3)	32 (9.8%)	25 (13.2%)	
Manufacturing	188 (11.4)	128 (11.3%)	45 (13.7%)	15 (7.9%)	
Other services (except public administration)	184 (11.2)	125 (11.0%)	39 (11.9%)	20 (10.5%)	
Retail, wholesale trade	137 (8.3)	86 (7.6)	35 (10.7%)	16 (8.3%)	
Educational services	147 (8.9)	113 (10.0%)	23 (7.0%)	11 (5.8%)	
Accommodation/food services/arts/entertainment	127 (7.7)	82 (7.3%)	32 (9.8%)	13 (6.8%)	
Public administration	98 (5.9)	67 (5.9%)	18 (5.5%)	13 (6.8%)	
Other	46 (2.8)	33 (2.9)	7 (2.1%)	6 (3.2%)	
Nature of injury, *n* (%)					
Sprain, strain, or dislocation	817 (49.5)	563 (49.7)	152 (46.3)	102 (53.7)	0.1283
Fracture	223 (13.5)	158 (14.0)	46 (14.0)	19 (10.0)	
Superficial or open wound	212 (12.9)	152 (13.4)	41 (12.5)	19 (10.0)	
Internal injury	258 (15.6)	178 (15.7)	51 (15.6)	29 (15.3)	
Other	65 (3.9)	38 (3.4)	20 (6.1)	7 (3.7)	
Unknown	75 (4.6)	43 (3.8)	18 (5.5)	14 (7.4)	
Prevalence of chronic conditions, pre-injury					
Back problems, *n* (%)	290 (17.8)	189 (17.0%)	69 (21.2%)	32 (17.8%)	0.2047
Arthritis, *n* (%)	269 (16.5)	182 (16.3%)	50 (15.5%)	37 (19.6%)	0.4548
Migraine, n (%)	275 (16.8)	185 (16.4%)	52 (16.1%)	38 (20.2%)	0.3991
Mood disorder current, *n* (%)	188 (11.5)	100 (8.9%)	67 (20.7%)	21 (11.5%)	< 0.0001
High blood pressure, *n* (%)	255 (15.6)	202 (17.9%)	41 (12.7%)	12 (6.5%)	0.0001

*Information on household income missing for 188 participants

Cannabis use was higher among participants less than 40 years of age, among men, and among workers in the construction, utilities, and mining sector, and lower among participants who were born outside of Canada (Table 1). Cannabis use was also higher among participants reporting not currently working at the 18-month interview, those reporting fair or poor self-related physical health and self-rated mental health, and those with K6 mental health symptom scores greater than 12 (Table 2).

**Table 2:** OLAWIS cohort, return to work, and recovery status at 18-month follow-up, by past-year cannabis use

	All respondents (*n* = 1650)	Past-year cannabis use			
		No use	Non-condition-related use	Condition-related use	*P* value
		(*N* = 1132)	(*N* = 328)	(*n* = 190)	
Past-year frequency of cannabis use					
Less than 3 times a month	NA	NA	166 (50.9)	65 (34.2)	0.0003
Once a week, less than daily	NA	NA	85 (26.1)	55 (29.0)	
Every day	NA	NA	75 (23.1)	70 (36.8)	
Medical authorization for cannabis use	NA	NA	65 (20.0)	46 (24.2)	0.2622
Pain severity, *n* (%)					
No pain	401 (24.4%)	296 (26.3%)	91 (27.8%)	14 (7.4%)	
Mild pain, functional impairment unlikely	685 (41.7%)	480 (42.6%)	154 (47.1%)	51 (26.8%	
Severe pain, functional impairment likely	557 (33.9%)	350 (31.1%)	82 (25.1%)	125 (65.8%)	
Employment status,* n* (%)					
Working at injury employer	987 (59.8)	728 (64.3)	170 (51.8)	89 (46.8)	< 0.0001
Working at different employer	269 (16.3)	159 (14.1)	81 (24.7)	29 (15.3)	
Not currently working	394 (23.9)	245 (21.6)	77 (23.5)	72 (37.9)	
Current WSIB services, *n* (%)	288 (17.5)	206 (18.3)	30 (9.2)	52 (27.7)	< 0.0001
Current health care for injury, *n* (%)	456 (31.2)	304 (30.3)	63 (22.3)	89 (50.0)	< 0.0001
Stressful healthcare experience (%)					
Not at all stressful	871 (59.4)	621 (61.8)	174 (61.3)	76 (42.9)	< 0.0001
Not very stressful	221 (15.1)	151 (15.0)	47 (16.6)	23 (13.0)	
A bit stressful	214 (14.6)	135 (13.4)	34 (12.0)	45 (25.4)	
Quite a bit stressful	93 (6.3)	57 (5.7)	16 (5.6)	20 (11.3)	
Extremely stressful	67 (4.6)	41 (4.1)	13 (4.6)	13 (7.3)	
Prescription opioid use (past year), *n* (%)	310 (19.1)	201 (18.2)	49 (15.0)	60 (31.8)	< 0.0001
Poor/fair physical health, *n* (%)	444 (26.9)	285 (25.2)	92 (28.1)	67 (35.3)	0.0131
Poor/fair mental health, *n* (%)	470 (28.5)	272 (24.1)	106 (32.3)	92 (48.7)	< 0.0001
K6 Score > 12, proportion (SD)	235 (14.5)	137 (12.3)	54 (16.6)	44 (23.3)	0.0002
Financial difficulties during work absence, *n* (%)					
Yes	784 (47.9)	520 (46.3)	137 (42.2)	127 (67.2)	< 0.0001

The prevalence of pain symptom severity 18 months post-injury was equivalent in the two OLAWIS cohorts: 24.4% of participants reported no pain, 41.7% reported mild pain, and 33.9% reported severe pain with functional impairment (Table 2). Condition-related cannabis use was strongly associated with severe pain symptoms: more than 65% of cohort members reporting condition-related cannabis use also reported severe pain symptoms at 18 months.

Among the 20% of cohort participants reporting past-year non-condition-related cannabis use, average wage replacement benefits ($5,613) and health care benefits ($4,288) were lower than average benefit expenditures for participants reporting no cannabis use ($7,764 and $6,187, respectively) (Table 3). Conversely, among the 11% of participants reporting condition-related cannabis use, average wage replacement benefits ($11,065) and health care benefits ($9,460) were higher than the average benefit expenditure for participants reporting no cannabis use.

**Table 3:** Wage replacement benefit and health care benefit expenditures, by past-year cannabis use

	*N* (%)	Wage replacement benefits			Health care benefits		
		Mean (median)	SD	*P* value	Mean (median)	SD	*P* value
No cannabis use	1117	$7,764.8 (3,611.84)	$9,741.2		$6,187.8 (1,930.80)	$8,708.0	
Non-condition-related cannabis use	323	$5,613.3 (2,408.19)	$7,331.6	< 0.0001	$4,288.5 (1,101.85)	$6,568.3	0.0003
Condition-related cannabis use	182	$11,056.5 (5,512.48)	$13,854.4	< 0.0001	$9,460.7 (5,211.80)	$10,303.2	< 0.0001

Benefit expenditure values (in Canadian dollars) have been truncated above the 95^th^ percentile

Table 4 presents estimates from the sequence of OLS regressions on the log-transformed benefit expenditure measures, reporting coefficient values for cannabis use and for pain symptom severity. Non-condition-related cannabis use was associated with 30% lower wage replacement benefits (exp^(−0.337)^ = 0.71) and 40% lower health care benefit expenditures (exp^(−0.507)^ = 0.60) compared to participants reporting no cannabis use. Condition-related cannabis use was associated with 40% higher wage replacement benefits (exp^(0.338)^ = 1.40) and 100% higher health care benefit expenditures (exp^(0.694)^ = 2.00).

**Table 4:** Association of pain severity and cannabis use with wage replacement benefit and health care benefit expenditures

	Main effects only (*n* = 1615)		Main effects, adjusted^*^ (*n* = 1615)		Main effects, adjusted for covariates^†^ (*n* = 1550)	
	Coefficient	*P* value	Coefficient	*P* value	Coefficient	*P* value
Wage replacement benefits						
Intercept			7.820		7.357	
Pain severity (intercept: no pain)	7.756					
Mild pain, functional impairment unlikely	0.153	ns	0.145	ns	**0.193**	0.047
Severe pain, functional impairment likely	**0.548**	< 0.001	**0.487**	< 0.001	**0.481**	< 0.001
Cannabis use (intercept: no use)	8.034					
Non-condition-related use	** − 0.337**	< 0.001	** − 0.313**	0.002	** − 0.309**	0.002
Condition-related use	**0.338**	< .0001	0.196	ns	0.254	ns
Health care benefits						
Intercept			6.776		5.879	
Pain severity (intercept: no pain)	6.686					
Mild pain, functional impairment unlikely	**0.519**	< .001	**0.505**	< 0.001	**0.417**	0.002
Severe pain, functional impairment likely	**1.364**	< .001	**1.266**	< 0.001	**1.110**	< 0.001
Cannabis use (intercept: no use)	7.386					
Non-condition-related use	** − 0.507**	< 0.001	** − 0.450**	0.001	− 0.251	ns
Condition-related use	**0.694**	< 0.001	**0.345**	0.05	**0.433**	0.012

Multivariable linear regression, log of benefit expenditures. Significant coefficients presented in bold

^*^Unweighted regression models estimating both pain severity and cannabis use

^†^Unweighted regression models estimating both pain severity and cannabis use, adjusted for age, sex, nature of injury, opioid prescription, cohort, and the prevalence of pre-injury chronic conditions

Table 4 also presents estimates of the association of pain severity and benefit expenditures. Severe pain symptoms were associated with approximately 70% higher wage replacement benefits (exp^(0.548)^ = 1.73, column 1) and more than 250% higher health care benefits (exp^(1.36)^ = 3.89) compared to participants reporting no pain. There was no statistical association between mild pain symptoms and wage replacement benefit expenditures (*β* = 0.153, ns). Mild pain symptoms were associated with approximately 70% higher health care benefit expenditures (exp^(0.519)^ = 1.68) compared to participants reporting no pain.

The positive association of severe pain symptoms and the negative association of non-condition-related cannabis use with wage replacement benefits were not substantially modified in the sequential modelling reported in Table 4. However, in the fully adjusted model, there was no longer an association between condition-related cannabis use and wage replacement benefits (*β* = 0.254, ns).

Similarly, the positive association of mild and severe pain symptoms with health care benefits was not substantially modified in the sequential modelling in Table 4. In the fully adjusted model, condition-related cannabis use had a positive association with health care benefit expenditures (exp^(0.433)^ = 1.54), indicating health care benefit expenditure was approximately 50% higher among participants reporting condition-related cannabis use compared to participants who did not use cannabis. There was no association between non-condition-related cannabis use and health care benefit expenditures in the fully adjusted model (*β* =  − 0.251, ns). The application of sample weights, to adjust for the over-sampling of participants with longer duration disability episodes, did not substantially alter the associations reported in Table 4.

## Discussion

In this observational study, 22.4% of participants reporting severe pain symptoms reported cannabis use for the treatment of conditions arising from their work-related injury compared to 6.0% of participants reporting mild pain or no pain. In regression analyses adjusted for the severity of pain symptoms and covariates measured at cohort inception, wage replacement benefit expenditures were similar between participants reporting condition-related cannabis use and participants not using cannabis. In contrast, health care benefit expenditures were elevated among participants reporting condition-related cannabis use compared to participants not using cannabis. After adjusting for covariates related to wage replacement benefit expenditures, participants reporting cannabis use unrelated to conditions arising from their work-related injury had lower wage replacement benefit expenditures compared to participants not using cannabis and had health care benefit expenditures similar to participants who did not use cannabis.

How do the findings reported in this study of cannabis use and work disability benefit expenditures align to previous literature in this field? We have not identified any contemporary studies in North America that have described cannabis use and disability benefit expenditures in cohorts of adults disabled by a traumatic occupational or non-occupational injury. Unlike many jurisdictions in the United States, recreational cannabis use has been legalized in this setting since 2018 and represents an important context to assess the potential benefits and harms of cannabis use in recovery and return-to-work following disabling injury.

### Interpretation/implications

Although not without concerns about harms to health arising from acute impairment effects of cannabis use (Carnide et al., 2023) or impairments arising from cannabis use dependence, cannabis use may have relatively benign effects on role function and work productivity compared to alcohol, tobacco, or opioid use among working-age adults (Sorge et al., 2020). The evidence presented in this study of adults recovering from a work-related injury or illness does not find a substantial association of cannabis use with disability benefit expenditures and health care benefit expenditures that would suggest either concerning harm, or significant benefit.

One important observation from this observational cohort of workers experiencing a disability episode following work-related injury is the relevance of stratifying cannabis use by whether use is for self-reported therapeutic or non-therapeutic purposes. Participants reporting use for therapeutic purposes to manage conditions arising from a work-related injury had poorer health status at the 18-month interview, a much higher prevalence of severe pain symptoms and a different profile of disability benefit expenditures compared to participants reporting cannabis use for non-therapeutic purposes*.* That wage replacement benefits expenditures were lower for participants reporting use for non-therapeutic purposes compared to use for therapeutic purposes is consistent with the higher proportion of the former in employment at the 18-month interview (76.5% vs 62.1%), which may in part be due to the younger average age and higher educational attainment of participants reporting use for non-therapeutic purposes. That health care benefit expenditures were also lower for participants reporting use for non-therapeutic purposes compared to use for therapeutic purposes does not appear to be related to differences in the nature of the disabling injury. The very substantial difference in the prevalence of severe pain symptoms at the 18-month interview (25.1% vs 65.8%), however, is a plausible explanation for the higher health care benefit expenditures and the substantially poorer measures of physical and mental health in the latter at the 18-month interview.

On the basis of the analytic approach applied in this study, it is unclear why participants reporting cannabis use for non-therapeutic purposes had, on average, lower wage replacement benefits than participants not using cannabis. Adjustment for characteristics measured at cohort inception, including age, sex, nature of injury, and pre-injury chronic conditions, did not attenuate the observed association. Health and function characteristics measured at the 18-month interview did not appear to describe important differences between participants reporting non-therapeutic cannabis use and those not using cannabis.

### Strengths and limitations

There are important strengths in this observational study. The recruitment of workers disabled by a work-related injury or illness was drawn from a population sample frame. The sampling design intentionally oversampled participants with longer duration disability episodes to ensure sufficient statistical power to confidently estimate factors that are relevant for understanding the determinants of long duration disability episodes. The use of information on disability benefit expenditures provides substantial insight into variation in the severity of work disability that is not well explained by diagnostic information on the nature of injury (Sears et al., 2015, Fan et al., 2012). There are a number of limitations arising from the study design that recommend caution in the interpretation of study findings. In this cohort recruited to be representative of workers experiencing a disabling work-related injury or illness, characteristics of health, function, and employment status are heterogeneous. More detailed information on the duration of cannabis use, dose, and compound and participants’ perceptions of the indication for use may have strengthened interpretation. Readers should also be aware that the healthcare benefit expenditure information reported in this study excludes healthcare services funded by the universal health insurance plan in this setting. As noted earlier in this paper, cannabis use for the therapeutic management of pain may diminish pain symptoms and pain-related impairment (Safakish et al., 2020). Additionally, we did not have a measure of pain prior to injury and acknowledge the possibility that some study participants may already have experienced a high burden of pain symptoms prior to injury. However, we would note that all members of the cohort were actively employed at the time of the disabling injury, suggesting a low prevalence of functional impairment prior to injury. The single measure of pain and of cannabis use available in this study, pertaining both to pain severity and to cannabis use at 18 months post-injury, is insufficient to accurately understand this temporal relationship and clearly indicates the importance of longitudinal study designs with repeated measurements of pain symptoms, cannabis use, and disability benefit expenditures in future research. As final reminder of the limitations of an observational study, readers need to be cautious in confidently interpreting causal relationships between cannabis use and disability benefit expenditures described in this study.

## Conclusion

This study is one of a limited number of studies of the association between cannabis use and disability benefit expenditures in a representative sample of work disability episodes. The evidence presented in this study of working-age adults recovering from a work-related injury or illness does not find a substantial association of cannabis use with disability benefit expenditures and health care benefit expenditures that would suggest either concerning harm, or significant benefit. These findings contribute information to support decision making among clinicians and disability insurance authorities on the potential benefits and risks associated with cannabis use in settings that have legalized cannabis use.

## Contributions to knowledge

What does this study add to existing knowledge?

- Replicating established evidence that severe pain symptoms are a common consequence of traumatic injury, this study describes the frequency of cannabis use as a pain management therapy in a cohort of workers recovering from work-related injury or illness.
- The evidence presented in this study of working-age adults recovering from a work-related injury or illness does not find a substantial association of cannabis use with disability benefit expenditures and health care benefit expenditures that would suggest either concerning harm, or significant benefit.

What are the key implications for public health interventions, practice, or policy?

- The findings of this study will be relevant to disability insurance providers’ policy decisions concerning entitlement for therapeutic use of cannabis.

## Data Availability

Procedures to access data from this study are available through contacting the corresponding author (CAM). Proposals for collaborative analyses will be considered by the study’s investigator team. The study questionnaire can be provided by contacting the corresponding author (CAM).
